# The Impact of a Vestibular-Stimulating Exercise Regime on Postural Stability in People with Visual Impairment

**DOI:** 10.1155/2015/136969

**Published:** 2015-10-25

**Authors:** Ida Wiszomirska, Katarzyna Kaczmarczyk, Michalina Błażkiewicz, Andrzej Wit

**Affiliations:** Faculty of Rehabilitation, Józef Piłsudski University of Physical Education in Warsaw, Ulica Marymoncka 34, 00-968 Warsaw 45, Poland

## Abstract

The aim of the study was to assess the impact of a vestibular-stimulating exercise regime on postural stability in individuals with visual impairment. The study group consisted of 70 people, including 28 persons (15 female and 13 male) with visual impairment and 42 (21 female and 21 male) without visual impairment. Each individual in the group with visual impairment was medically qualified for a 3-month training program. The research methodology included medical examination, anthropometric tests, and stabilometry tests on a Biodex Balance System SD (BBS). The tests were conducted twice: once before the start of training and again after 3 months of rehabilitation. The group with visual impairment showed significantly worse postural stability results than the control group for most of the stability parameters evaluated (OSI, APSI, and MLSI). Differences were noted between the groups with and without visual impairment for dynamic tests in women and for static tests in men. After training, the two groups showed roughly similar results for the stabilometry test with eyes closed. We conclude that exercises stimulating the vestibular system with head and body movements should be recommended for individuals with visual impairments to achieve better balance retention.

## 1. Introduction

Balance in the human body, the stability of its characteristic upright postural position, is maintained, controlled, and monitored by a complex system, consisting of the vestibular organs, the visual organs, and the organs of deep perception, touch, and pressure. Nerve signals then reach the effector organs, such as the muscles of the torso, limbs, and eyes, provoking reflex reactions coordinating body posture [1]. Impaired postural stability may be caused by dysfunction in the integration of each of the four sensory systems: visual [2, 3], proprioceptor [4, 5], exteroceptor [6], and vestibular [7, 8].

Sight, in particular, has been found to play a key role [9–11] in maintaining postural stability. Visual impairment reduces the ability to maintain balance [12] and may cause frequent injury [2, 13], including falls, occupational injuries, and traffic-related injuries. A thorough review of the literature [14] established that the risk of injury due to falls is higher for those with visual impairment than for the general population. Studies comparing individuals with/without visual impairment undergoing static and dynamic tests have confirmed that about 80% of our sensory perception is gathered in such situations by the visual system. Our movements are mainly controlled and coordinated by the eyes. Hence not only is the visual system responsible for cognition of objects, it is also used to give information to the brain about the position of our body [15], which processes and integrates information from other sensory systems to select the strategy for maintaining balance [16].

Nevertheless, the literature contains inconclusive and limited results on the possibility of compensating for balance in individuals with visual impairment. Some studies have shown that persons who are blind can maintain balance better than individuals without visual impairment [17], while others have reported results pointing in the opposite direction [18, 19]. One reason for this may be because blind individuals are able to compensate to mitigate their disability: other elements involved in ensuring postural stability—deep sensation and the vestibular system—may function more actively in individuals who are blind in vertical posture maintenance and thus compensate for the lack of visual stimuli [2, 8]. Vestibular system function is the main source of such compensation [15], as vestibular and labyrinth reflexes play a special role when other sources are reduced or absent [8].

The absence of the sense of vision can affect the motor skills of the individual, although this does not prevent individuals who have visual impairment from being physically active. The practice of sporting activity can bolster their sense of independence and autonomy, generating multiple benefits of a physiological, sociological, and psychological nature. It is therefore important to find new ways to encourage social relations between individuals with visual impairment and their peers as well as other people outside their homes [20].

Various studies have reported the positive impact of vestibular rehabilitation on postural stability in individuals experiencing dizziness [21–23], as well as patients who have disorders such as multiple sclerosis (MS) [24]. A review [25] covering 27 studies involving 1668 individuals with unilateral peripheral dysfunction of the vestibular organ who had undergone vestibular rehabilitation—a distinctive form of therapy that aims to limit dizziness, balance disruptions, and improve gaze stability, postural stability, and general physical condition—concluded that such therapy is safe and effective in such cases. These findings suggest the potential viability of developing a similar experimental exercise program for individuals without such vestibular disorders.

The goal of our study, therefore, was to assess the impact of training meant to stimulate the vestibular system in individuals with visual impairment. We hypothesized that such training would improve the effective use of this system, creating new motor patterns and thereby improving postural stability. Such research may help us better understand the mechanisms involved in balance maintenance in persons with visual impairment and encourage the practice of proper exercise.

## 2. Method

### 2.1. Participants

A total of 70 people took part in the study, including 28 individuals with visual impairment as the experimental group and 42 individuals without visual impairment as the control group. The inclusion criteria for the experimental group were visual impairment from birth or in the first months of life, the absence of any disease affecting the sense of balance, and no previous performance of motor activity aimed at improving the vestibular system. Qualification was conducted by a medical doctor and included a medical history interview (on history of falls, fractures, stumbling, and dizziness), analysis of medical records, clinical examination, assessment of eye damage, electrocardiography (ECG) and assessment of the cranial nerves for any possible meningeal symptoms, cerebellar testing (finger-to-nose test, diadochokinesis, and pronator drift test), and the results of static and dynamic tests which assess the correctness of posture and gait (Romberg's test, Unterberger's test, the Babinski-Weil test, the Fukuda test, and the straight line test).

The participants with visual impairment were all adolescent pupils at a vocational school for the blind in Laski (Poland), categorized for total or legal blindness according to the US Association for Blind Athletes (USABA) sport classification: blind (B1s; 22) and low vision (B3s; 10). Twenty-four of the 32 adolescents were congenitally blind; the other eight experienced onset in early childhood.

The individuals with visual impairment who qualified for the training program were preliminarily divided by the qualifying physician into 2 groups based on health criteria: blind without damage to the vestibular system (20 patients) and low vision (i.e., able to sense light) without damage to the vestibular system (8 people). However, in view of the fact that there was no difference in stabilometric results between these groups (much like in the study of Haibach et al. [18]), we considered them together as a single “group with visual impairment.”

The members of the control group, in turn, selected on the basis of age and physical traits (height and weight), were either students of the Academy of Physical Education in Warsaw (majoring in physiotherapy, a course of study that does not involve any particularly intense regime of physical activity) or high school students, in whom the qualifying physician found no damaged sense of balance or postural disorders. This group did not participate in the vestibular training program.

The general level of physical activity was similar for both groups of participants (attending physical education classes three times a week for 45 minutes). Approval was obtained from the Institute's Research Ethics Commission and additional informed consent was obtained from all participants for whom any identifying information is included in this paper.

### 2.2. Measurements

Postural stability was measured using a Biodex Balance System SD (BBS), an instrument designed to measure and train postural stability on static or unstable surfaces. The BBS consists of a circular platform that is free to move in the anterior-posterior and medial lateral axes simultaneously, offering the ability to control the movement degree of the platform by 12 levels. The BSS device is interfaced with dedicated software (Biodex Medical Systems, Inc., version 1.3.4), allowing the BSS to measure the degree of tilt in each axis, providing an average sway score. Eight springs located underneath the outer edge of the platform provide resistance to movement (stability level of the platform), with resistance levels ranging from 12 (most stable) to 1 (least stable). The BBS has a display to give feedback in real time about the posture and is calibrated before use.

The participants stood on the BBS supported on their two legs, facing towards the display, for all time trials. All trials were conducted without shoes and foot position was recorded using coordinates on the platform's grid to ensure the same stance and, therefore, consistency with future tests. In this research three measurement protocols were used: the Postural Stability Test (PST) under two different conditions (stable platform, unstable platform level 8) and the Fall Risk Test (FRT) with eyes closed. The unstable platform level 8 was chosen for our purposes after a pilot study indicated that this would be an appropriate level to test for dynamic balance, not too difficult for most of the participants. In the FRT, the platform is unstable and thus permits investigators to obtain the fall risk index (FRI). This test was conducted using the standard software configuration: three trials of 20 seconds each, with ten seconds rest between tests, and platform levels varying from 12 to 6. In the PST the platform is static in the anterior-posterior stability index (APSI) and medial-lateral stability index (MLSI) axes and can measure the overall stability index (OSI). This test consisted of three trials, each 20 seconds long, with one minute between trials. These indexes represent fluctuations around a zero point established prior to testing when the platform was stable [26].

For all participants, basic somatic measurements were taken—body weight and height using a standard electronic scale and anthropometer. On the basis of these anthropometric measurements the Body Mass Index (BMI) was calculated. For participants with visual impairment, measurements were performed twice—once before training and again immediately after the training cycle.

The recorded data were analyzed statistically using the STATISTICA software (v.10). A 2 (group) × 2 analysis of variance (ANOVA) was performed with repeated measures on the OSI, APSI, MLSI, and FRI tests, for significant differences between the groups and pretraining baseline measures. Significant group-by-test interactions were further broken down by comparing pretraining differences separately for each group using Tukey's post hoc HSD test for differing sample sizes. Normality of distribution was analyzed by the Shapiro-Wilk test. Because the tested features have normal distributions, variables were compared between the first and second test using parametric Student's *t*-test (*p* < 0.05).

### 2.3. Intervention

The group of participants with visual impairment underwent training sessions (approximately 20 minutes each), twice a week, over the course of 3 months, with the aim of encouraging them to engage in more physical exercise, improve their physical fitness, and particularly improve their balance control. In particular, stimulation of the vestibular organ [27] was achieved by having these participants rotate their heads and bodies in the sagittal, transverse, and frontal planes.

In designing this exercise program, we anticipated that, by triggering sensory conflict, such activity would force the correct responses from the vestibular and proprioceptor organs, leading to an improved relationship between them, which in turn should improve balance control. Stimulation caused by movements of the head and body is perceived as a nonspecific impulse. When the stimuli are repeated regularly and not followed by a loss of balance, the response is inhibited, which in turn leads to the formation of a new image of the vestibular position in the CNS. This method aims to improve the perception and verification of information originating from the external and internal environment.

The exercises started with gentle, slow movements of the head without visual control in various low positions (lying position), such as body bent at 30°, with participants lying on their backs or sides. For example, after first lying on their back, participants shifted to lying on their right side and then to their left side, repeating this approximately 10 times (in keeping with their individual capabilities). After all the repetitions were performed, participants stopped moving for approximately 3 minutes while lying on their right side. The cycle was then repeated, this time stopping on the left side. Another exercise involved sitting with legs extended straight and then performing side bends to rest the arms on the mat behind the body, alternatingly on the left and right side. After several repetitions were performed, movement was halted for about three minutes in the side-bend position with upper limbs bent and with head held motionless, alternatingly on the left side and the right side. Following relaxation to music, the next exercise involved gentle, slow movements of the head with the participants keeping their eyes closed and maintaining the final position for approximately 3 minutes. Once in the final positions, participants performed breathing and relaxation exercises with their heads kept motionless. Such exercises without visual control, supplemented with additional auditory impulses (music), aimed to improve kinaesthesia by stimulating signals from the vestibular organ through head and body movements. In the final phase of each movement, the participant was stopped in the corrective position and asked to maintain the set position. The purpose of this halting of movement for about three minutes after performing each simple exercise movement was to equalize the flow (following head movement) of endolymph in the semicircular canals and the otoliths of the saccule and utricle of the vestibular organ. As the exercise program progressed, the number of repetitions was gradually increased and the support plane was reduced. These exercises were further supplemented with additional auditory stimuli (soothing music), intended to help create an appropriately positive mood, calm the subjects, and make the motor exercises more attractive. The relevance of such music in attempting to boost vestibular performance may be justified by the close proximity and close interrelatedness of the auditory and vestibular organs [28] and the fact that receptor cells for the both organs are situated in the inner ear and transmit information along the same, eighth cranial nerve (the vestibulocochlear nerve) [29]. The maximum heart rate during exercise did not exceed 100 bpm (a fact that underscores the low intensity of the exercise regime).

## 3. Results

No significant differences were found (Tukey's post hoc HSD test for different sample sizes) among participants of the same sex with respect to age, body height, weight, and Body Mass Index (BMI) (Table 1). But because there were significant statistical differences found in traits such as body height and weight between participants of different sexes, the male and female groups were considered separately, thus yielding four subgroups (with/without visual impairment, male/female). According to the BMI standards adopted by the WHO [30], the arithmetic mean of this variable for each group fell within the normal range.

The training program was completed by 13 men and 15 women in the group with visual impairment. Four participants were excluded from the study because of illness-related absences, due to an adopted rule whereby participants were excluded from the program if they missed two sessions.

Tables 2 and 3 show the stabilometric parameters obtained in the first test by the group with visual impairment as compared to the control group. No statistically significant differences were noted in these tests between the sexes (comparing group 1 to group 3 and group 2 to group 4). No significant differences were observed in any of the static parameters for women under closed-eye conditions, while men performing the same test did show significantly worse results in the group with visually impairment than the control group. In unstable-platform tests, women with visual impairment fared worse than women without visual impairment, except in the ML index.

For the men with visual impairment, there were no significant differences in the unstable platform protocol test; however, this test was not performed for 30% of the respondents (*n* = 5), for whom it proved too difficult and who were excluded from the study as a result.

Analysis of the average values for the overall stability index after training showed statistically significant differences for both women with visual impairment (Table 4), with a value of 2.17 ± 0.89 to 1.43 ± 0.75, and men with visual impairment (Table 5), with a value of 2.58 ± 1.5 to 1.42 ± 0.73, although the OSI derivatives, the AP and ML parameters, do not show the same changes for the two genders.

In women there are no changes, whereas among men the AP index significantly improved. A similar pattern was found for the dynamic test, with the OSI changing significantly at the *p* < 0.01 level in individuals of both sexes and the AP index improving only in women. Noteworthy progress was made on the FRT protocol (platform levels varying from 12 to 6) by both groups. The test using variable platform stability settings is therefore the most difficult test for dynamic postural stability.

After the training cycle was completed, the results were compared between the participants in the groups with and without visual impairment, finding no statistically significant differences between the groups of the same sex in closed-eye conditions. Women without visual impairment had a general stability index in the static test of 1.9 ± 0.78, whereas women with visual impairment after training showed 1.43 ± 0.75; for men the respective values were 1.42 ± 0.61 and 1.42 ± 0.73. For the dynamic test, the stability indexes also did not differ significantly between the group of women without visual impairment (2.24 ± 0.48) and with visual impairment after training (2.67 ± 0.6), or men without visual impairment (3.59 ± 1.69) and men with visual impairment after training (3.25 ± 0.96).

## 4. Discussion 

Balance is an indispensable factor for individuals with visual impairment, helping to encourage their integration in space [20]. Balance disorders have been the subject of numerous studies, and the results of these have been used in developing forms of exercise designed to address such disorders. Studies have shown that persons with visual impairment perform worse on Postural Stability Tests than those without visual impairment [28–30].

In the study reported herein, prior to training, no significant differences were noted between the two groups of women in static tests, whereas in unstable-platform tests such differences are observed in the general index and in anterior-posterior deviations. Male participants without impaired vision, on the other hand, were found to differ significantly in terms of the stabilometric parameters with the static tests. In tests on an unstable platform (level 8), without visual control, men in the group with visual impairment did not differ significantly from men in the group without visual impairment. This may be due to the fact that 5 men were unable to perform the unstable-platform test (some individuals with visual impairment have trouble maintaining balance even on a slightly unstable base, and these individuals were excluded from the study as a result).

After the training regime, however, the results of participants (men and women) with impaired vision became approximate to those of participants without impaired vision operating with eyes closed and did not differ significantly in terms of any of the parameters evaluated. Overall, then, our study indicates that individuals with impaired vision can significantly improve their balance, although it was found that even specialized training is not capable of fully compensating for the impact of vision loss on balance retention. Different results in stability measurements between blind and sighted individuals (in tests with eyes closed) were obtained by Melzer et al. [31]. They evaluated stability in static tests with no additional auditory-memory tasks and found no differences between the groups. In contrast, when their participants performed additional auditory-memory tasks, in sighted participants the stability parameters dropped, in contrast to participants who were blind. This may suggest that individuals with/without visual impairment use different strategies to maintain stability.

Giagazoglou et al. [11] presented results that differed from those of the above authors but which agreed with the results in our study. They found that the ability to control balance in both the AP and ML directions was significantly worse in female participants who were blind than in those who were fully sighted. When sighted women performed the tests blindfolded, their COP deviations increased significantly in both directions; however, the blind participants still consistently achieved significantly inferior results. The results of Blomqvist and Rehn's [9] study using the DOLS (Dynamic One-Leg Stance) test, in turn, indicate that individuals who are blind are not able to compensate for loss of vision by utilizing other sensory extravisual information during dynamic motor tasks on the right and left limb.

Aydog et al. [32] found no differences between the results of individuals playing goalball who are blind and sighted, in all three indices (SI) on the BBS platform. The athletes' postural stability was better than that of blind people who engaged in little physical activity. Specific exercise, in this case playing goalball, causes adaptation in the form of greater system tolerance to imbalances. In Colaka et al.'s study [20] goalball players showed a significant advantage over their respective control groups on the Flamingo balance test. The better result of goalball players on the balance test perhaps suggests that the training program may exert an impact on motor skills [20]. Visual impairment need not be a factor differentiating predispositions towards tolerating imbalances in the body. This is confirmed by the findings of Marini et al. [33], which found persons who are blind practicing a variety of Italian basketball to be in better control of postural stability than those not doing so, as evaluated by functional tests (Fukuda and Tinetti).

The positive effects of exercise (better results of stabilometric parameters) have been repeatedly demonstrated. The authors of many studies have sought to identify an optimal exercise program for improving balance in order to minimize the future risk of falls. Gioftsidou et al. [34] used the BBS platform to study the effect of one year of football training on maintaining balance. The authors observed no statistically significant changes. Our study, in turn, provides evidence that specially designed training, dominated by balance exercises, must be conducted for postural control to be improved.

In our study, individuals with visual impairment participated in an easily implemented training program which actuates the vestibular system through motions of the head and body in all planes. This proposed form of activity has the advantage of not requiring great physical exertion, not involving a wide range of abilities, and predominantly involving relaxation exercises that also stimulate cognitive functions. It therefore can serve to activate individuals with visual impairment who do not engage in sporting activity for physical exercise; the relatively easy activity may also yield positive results in terms of improved balance and also other benefits of a psychological and sociological nature, through group work and integration. Our results showed encouraging improvements in the overall stability parameters in all test protocols used. A positive effect of vestibular rehabilitation on improving postural stability has been found in patients with dizziness [21–23] and in patients with multiple sclerosis [24].

The assumption of all these studies is that vestibular-stimulating physical activity triggers sensory conflicts which cause appropriate stimulation of the vestibular system and proprioceptors, leading to improved connections between them, which we assumed would compensate for the blindness and which in turn would improve the postural stability. Repeated linear and angular movements are sensed as a nonspecific stimulus, but when these operations are repeated regularly and there is no danger of losing balance because of them, this reaction is impeded, leading to the formation in the central nervous system of a new image of the vestibular situation.

Morozetti et al. [35] showed that rehabilitation for dizziness consisting of specific movements of the head resulted in a significant improvement in otoneurological clinical evaluation and in participants' perceptions of the head. Their test group obtained an improvement in balance assessed by the overall index of stability, while the anterior-posterior and mediolateral indices did not always differ significantly from the initial data, although reductions in the average were noted. Vestibular and labyrinth reflexes play a special role when other sources are reduced or absent [8]. Thus, the observed progress is important because stability decreases together with the lack of visual control. Radvay et al. [36] also observed a greater decrease in the stability results in dynamic tests, as confirmed by our own study presented in this paper.

In closing, we conclude that an appropriately designed regime of simple exercise can improve balance in individuals with visual impairment, although of course the lack of visual sensitivity in these patients cannot be fully replaced by activation of other receptors or nerve pathways.

## 5. Limitations

Two specific points bear mentioning here: firstly, the control group did not participate in the training program and were not also tested for posturography after three months. Secondly, 30% of the participants did not complete the unstable-platform test because they fell off, being unable to maintain balance in three attempts of 20 seconds each. Both of these factors could impact the interpretation of the results.

In the more general sense, we should also point out that the regime tested herein, although performed in group exercises, might be seen as less social or well-known than the reviewed and compared interventions involving football, goalball, and so forth. As such, future research may look into how similar results can be achieved through a more social, functional intervention.

## 6. Implications for Practitioners

Exercises designed to stimulate the vestibular system through head and body movements should be recommended for patients with visual impairment to achieve better balance retention, although a congenital dysfunction of vision cannot be fully compensated for by activating other neural pathways.

## Figures and Tables

**Table 1:** Participants' demographics (mean ± SD).

Group	*n*	AGE [years]	Body height [cm]	Body mass [kg]	BMI
Female 1 (without visual impairment)	21	20.0 ± 0.89	163.14 ± 3.26	57.43 ± 4.51	21.58 ± 1.83
Female 2 (with visual impairment)	15	19.14 ± 1.56	159.4 ± 6.45	53.64 ± 9.15	21.14 ± 3.45
Male 3 (without visual impairment)	21	18.85 ± 1.35	175.43 ± 2.56	69.18 ± 6.22	23.01 ± 2.85
Male 4 (with visual impairment)	13	19.08 ± 1.6	169.6 ± 6.57	62.73 ± 12.46	21.86 ± 4.82

**Table 2:** Female participants' baseline measures for stability index (mean ± SD): women without (female 1) and with (female 2) visual impairment.

Stability index (SI)	Female 1 (*n* = 21)	Female 2 (*n* = 15)	“Effect Size”	*p*
OSI CE, static	1.9 ± 0.78	2.17 ± 0.89	0.32	“NS”
APSI CE, static	1.39 ± 0.83	1.45 ± 0.74	0.08	“NS”
MLSI CE, static	0.99 ± 0.59	1.35 ± 0.77	0.53	“NS”
OSI CE, 8	2.24 ± 0.48	3.28 ± 0.59	**1.94**	***p ***<0.033
APSI CE, 8	1.57 ± 0.42	2.57 ± 0.68	**1.82**	***p ***<0.004
MLSI CE, 8	1.3 ± 0.22	1.58 ± 0.42	0.87	“NS”

OSI: overall stability index, APSI: anterior-posterior stability index, MLSI: medial-lateral stability index, OE: open eyes for participants fully sighted, closed for those with visual impairment, CE: closed eyes, static: stable platform, 8: dynamic balance level 8, and “Effect Size”: mean of female 2 − mean of female 1/standard deviation.

*p*: Tukey's HSD test.

**Table 3:** Male participants' baseline measures for stability index (mean ± SD): men without (male 3) and with (male 4) visual impairment.

Stability index (SI)	Male 3 (*n* = 21)	Male 4 (*n* = 13)	“Effect Size”	*p*
OSI CE, static	1.42 ± 0.61	2.58 ± 1.5	**1,09**	**0.000**
APSI CE, static	1.13 ± 0.57	1.72 ± 1.06	**0,72**	**0.049**
MLSI CE, static	0.66 ± 0.36	1.48 ± 1.5	**0,88**	**0.002**
OSI CE, 8	3.59 ± 1.69	3.67 ± 0.89	0,08	“NS”
APSI CE, 8	2.24 ± 1.01	2.65 ± 0.71	0,48	“NS”
MLSI CE, 8	2.3 ± 1.18	1.99 ± 0.79	−0,31	“NS”

OSI: overall stability index, APSI: anterior-posterior stability index, MLSI: medial-lateral stability index, OE: open eyes for participants fully sighted, closed for those with visual impairment, CE: closed eyes, static: stable platform, 8: dynamic balance level 8, and “Effect Size”: mean of male 4 − mean of male 3/standard deviation.

*p*: Tukey's HSD test.

**Table 4:** Comparison of pre- and postexercise values for stability index (mean ± SD) in women with visual impairment.

Stability index (SI)	Female 2 preexercise (*n* = 15)	Female 2 postexercise (*n* = 15)	“Effect Size”	*p*
OSI CE, static	2.17 ± 0.89	1.43 ± 0.75	0.9	0.016
APSI CE, static	1.45 ± 0.74	1.11 ± 0.77	0.45	“NS”
MLSI CE, static	1.35 ± 0.77	0.88 ± 0.58	0.7	“NS”
OSI CE, 8	3.28 ± 0.59	2.67 ± 0.6	1.02	0.003
APSI CE, 8	2.57 ± 0.68	1.96 ± 0.51	1.02	0.007
MLSI CE, 8	1.58 ± 0.42	1.41 ± 0.5	0.37	“NS”
FRI (12–6)	2.83 ± 0.45	2.37 ± 0.45	1.02	0.029

*Note*. OSI: overall stability index, APSI: anterior-posterior stability index, MLSI: medial-lateral stability index, FRI: fall risk index, CE: closed eyes, 8: dynamic balance level 8, and static: platform stable.

*p*: Student's *t*-test.

**Table 5:** Comparison of pre- and postexercise values for stability index (mean ± SD) in men with visual impairment.

Stability index (SI)	Male 4 preexercise (*n* = 13)	Male 4 postexercise (*n* = 13)	“Effect Size”	*p*
OSI CE, static	2.58 ± 1.5	1.42 ± 0.73	**1.04**	**0.008**
APSI CE, static	1.72 ± 1.06	1.0 ± 0.56	**0.89**	**0.048**
MLSI CE, static	1.48 ± 1.5	0.83 ± 0.49	0.65	“NS”
OSI CE, 8	3.67 ± 0.89	3.25 ± 0.96	**0.44**	**0.006**
APSI CE, 8	2.65 ± 0.71	2.3 ± 0.8	0.46	“NS”
MLSI CE, 8	1.99 ± 0.79	1.83 ± 0.62	0.23	“NS”
FRI (12–6)	3.35 ± 0.72	2.71 ± 0.65	**0.93**	**0.002**

*Note*. OSI: overall stability index, APSI: anterior-posterior stability index, MLSI: medial-lateral stability index, FRI: fall risk index, CE: closed eyes, 8: dynamic balance level 8, and static: platform stable.

*p*: Student's *t*-test.
